# Processing of Polymer Nanocomposites Reinforced with Polysaccharide Nanocrystals

**DOI:** 10.3390/molecules15064111

**Published:** 2010-06-08

**Authors:** Alain Dufresne

**Affiliations:** The International School of Paper, Print Media and Biomaterials (Pagora), Grenoble Institute of Technology, BP 65 - 38402 Saint Martin d'Hères Cedex, France; E-Mail: Alain.Dufresne@pagora.grenoble-inp.fr; Tel.: +33-4-76-82-69-95; Fax: +33-4-76-82-69-33

**Keywords:** cellulose, nanocomposites, processing, nanocrystals, whiskers, microfibrils

## Abstract

Aqueous suspensions of polysaccharide (cellulose, chitin or starch) nanocrystals can be prepared by acid hydrolysis of biomass. The main problem with their practical use is related to the homogeneous dispersion of these nanoparticles within a polymeric matrix. Water is the preferred processing medium. A new and interesting way for the processing of polysaccharide nanocrystals-based nanocomposites is their transformation into a co-continuous material through long chain surface chemical modification. It involves the surface chemical modification of the nanoparticles based on the use of grafting agents bearing a reactive end group and a long compatibilizing tail.

## 1. Introduction

The potential of nanocomposites in various sectors of research and application is promising and is attracting increasing investments. In the nanocomposite industry, a reinforcing particle is usually considered as one where at least one of its linear dimension is smaller than 100 nm. Due to the hierarchical structure and semicrystalline nature of polysaccharides (cellulose, starch and chitin), nanoparticles can be extracted from these naturally occuring polymers. Native cellulose and chitin fibers are made up of smaller and mechanically stronger long thin filaments, called microfibrils, consisting of alternating crystalline and non-crystalline domains. Multiple mechanical shearing actions can be used to release more or less individually these microfibrils. When using cellulose this material is usually called microfibrillated cellulose (MFC). Longitudinal cutting of these microfibrils can be performed by submitting the biomass to a strong acid hydrolysis treatment, allowing dissolution of amorphous domains. The ensuing nanoparticles occur as rod-like nanocrystals or whiskers. Similar acidic treatment carried out on starch granules allows obtaining platelet-like nanoparticles.

The impressive mechanical properties and reinforcing capability, abundance, low weight, and biodegradability of cellulose nanocrystals make them ideal candidates for the processing of polymer nanocomposites [1,2,3,4]. With a Young’s modulus of over 100 GPa and a surface area of several hundred m^2^·g^-1^ [5], they have the potential to significantly reinforce polymers at low filler loadings. A broad range of applications of nanocellulose exist, even if a high number of unknowns remain to date. Tens of scientific publications and experts show its potential, even if most of the studies have focused on the mechanical properties as a reinforcing phase and the liquid crystal self-ordering properties. However, as for any nanoparticle, the main challenge is related to their homogeneous dispersion within a polymeric matrix.

Polysaccharide nanoparticles are obtained as aqueous suspensions and most investigations have focused on hydrosoluble (or at least hydrodispersible) or latex-form polymers. The dispersion of these nanocrystals in non-aqueous media is possible using surfactants or chemical grafting and it opens other possibilities for nanocomposite processing. Polysaccharide nanocrystals possess a reactive surface covered with hydroxyl groups, providing the possibility of extensive chemical modification. Although this strategy decreases the surface energy and polar character of the nanoparticles, improving by the way the adhesion with non polar polymeric matrix a detrimental effect is generally reported for the mechanical performances of the composite. This unusual behavior is ascribed to the originality of the reinforcing phenomenon of polysaccharide nanocrystals resulting from the formation of a percolating network thanks to hydrogen bonding forces. Therefore, grafting of long chains instead of small molecules can be used to preserve the mechanical properties of the material. Very few studies have been reported concerning the processing of polysaccharide nanocrystals reinforced nanocomposites by extrusion methods. This paper reviews the different processing techniques of polysaccharide (mainly cellulose) nanocrystal-reinforced polymer nanocomposites.

## 2. Results and Discussion

### 2.1. Polymer latexes

This mode of processing of nanocomposite materials allows preservation of the individualized state of the nanoparticles resulting from their colloidal dispersion in water. The stability of the aqueous suspensions depends on the dimensions, size polydispersity and surface charge of the dispersed species. The use of sulfuric acid to prepare nanocrystals leads to more stable aqueous suspensions than those prepared using hydrochloric acid [6,7]. It was shown that the H_2_SO_4_-prepared nanoparticles present a negatively charged surface, while the HCl-prepared nanoparticles are not charged. During acid hydrolysis via sulfuric acid, acidic sulfate ester groups are likely formed on the nanoparticle surface. This creates an electric double layer repulsion between the nanoparticles in suspension, which plays an important role in their interaction with a polymer matrix and with each other.

**Figure 1:**
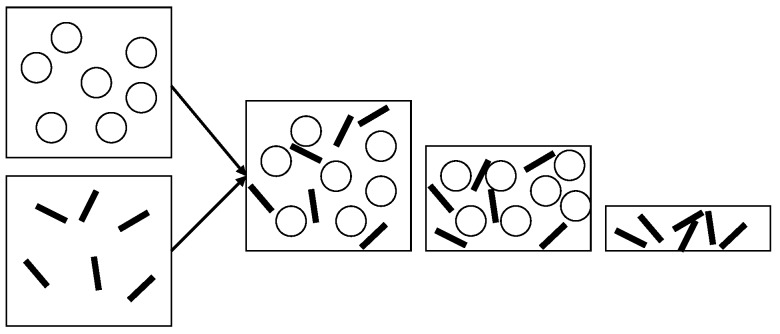
Processing of polysaccharide nanocrystals reinforced polymer nanocomposite films using polymer latex.

The first publication reporting the preparation of polysaccharide nanocrystals reinforced polymer nanocomposites was carried out using a latex obtained by copolymerization of styrene and butyl acrylate [poly(S-co-BuA)] and tunicin (the cellulose extracted from a tunicate – a sea animal) whiskers [8]. The same copolymer was used in association with wheat straw [9,10] or sugar beet [11] cellulose nanocrystals, potato starch nanocrystals [12,13], and squid pen [14] and *Riftia* tubes chitin whiskers [15]. Other latexes such as poly(β-hydroxyoctanoate) (PHO) [16,17,18], polyvinylchloride (PVC) [19,20,21,22], waterborne epoxy [23], natural rubber (NR) [24,25,26,27,28,29,30], and polyvinyl acetate (PVAc) [31] were also used as matrix. Recently, stable aqueous nanocomposite dispersions containing cellulose whiskers and a poly(styrene-co-hexyl-acrylate) matrix were prepared via miniemulsion polymerization [32]. Addition of a reactive silane was used to stabilize the dispersion. Solid nanocomposite films can be obtained by mixing and casting the two aqueous suspensions followed by water evaporation performed above the glass transition temperature of the polymer as shown in Figure 1. During water evaporation, the solid content in the medium increases and the latex particles get closer. When getting in touch each other these soft polymeric particles deform and adopt a polyhedral form. Boundary between the former particles disappears by chain diffusion leading to a continuous polymer film containing the dispersed polysaccharide nanoparticles.

Alternative methods consist of freeze-drying and hot-pressing or freeze-drying, extruding and hot-pressing the mixture. The dispersion of nanoparticles in the nanocomposite film strongly depends on the processing technique and conditions. Scanning electron microscopy (SEM) comparison between either cast and evaporated or freeze-dried and subsequently hot-pressed composites based on poly(S-co-BuA) reinforced with wheat straw whiskers, demonstrated that the former were less homogeneous and displayed a gradient of whiskers concentration between the upper and lower faces of the composite film [9,10]. This sedimentation phenomenon was confirmed using wide angle X-ray scattering (WAXS) by comparing the X-ray beams diffracted by the two faces. It was suggested that this observation was most probably induced by the processing technique itself and that casting/evaporation technique results in the less homogenous films, where the whiskers have tendency to orient randomly into horizontal plans.

The polymeric particle size seems to play a predominant role [16]. Larger latex particle size results in higher mechanical properties. Indeed, the polymeric particles act as impenetrable domains to polysaccharide nanoparticles during the film formation due to their high viscosity. Increasing latex particle size leads to an increase of the excluded volume and these geometrical constraints seem to affect the whiskers network formation.

### 2.2. Hydrosoluble or hydrodispersible polymers

After dissolution of the hydrosoluble or hydrodispersible polymer, the aqueous solution can be mixed with the aqueous suspension of polysaccharide nanocrystals. The ensuing mixture is generally evaporated to obtain a solid nanocomposite film. It can also be freeze-dried and hot-pressed. The preparation of polysaccharide particles with reinforced starch [33,34,35,36,37,38,39,40,41,42], silk fibroin [42], poly(oxyethylene) (POE) [43,44,45,46,47], polyvinyl alcohol (PVA) [48,49,50,51,52], hydroxypropyl cellulose (HPC) [48,49], carboxymethyl cellulose (CMC) [53] or soy protein isolate (SPI) [54,55] has been reported in the literature. New nanocomposites with waterborne polyurethane (WPU) as a matrix were also prepared by filling with a low loading of starch nanocrystals (StNs) as a nano-phase [56]. The resulting materials possessed enhanced strength, elongation and Young’s modulus. The chemical grafting of the StNs did not affect positively the strength and elongation, because such a treatment inhibited the formation of physical interaction and increasing network density in nanocomposites.

### 2.3. Non aqueous systems

Except for the use of an aqueous polymer dispersion, or latex, an alternative way to process non-polar polymer nanocomposites reinforced with polysaccharide nanoparticles composites consists in their dispersion in an adequate (with regard to matrix) organic medium. Coating with a surfactant or surface chemical modification of the nanoparticles can be considered. The global objective is to reduce their surface energy in order to improve their dispersibility/compatibility with non-polar media.

Coating of cotton and tunicin whiskers by a surfactant such as a phosphoric ester of polyoxyethylene (9)-nonyl phenyl ether was found to lead to stable suspensions in toluene and cyclohexane [57] or chloroform [58]. Coated tunicin whiskers reinforced atactic polypropylene (aPP) [59], isotactic polypropylene (iPP) [60], or poly(ethylene-co-vinyl acetate) (EVA) [61] were obtained by solvent casting using toluene. The same procedure was used to disperse cellulosic nanoparticles in chloroform and process composites with poly lactic acid (PLA) [58,62]. Nanocomposite materials were also prepared by dispersing cellulose acetate butyrate (CAB) in a dispersion of topochemically trimethylsilylated bacterial cellulose nanocrystals in acetone and subsequent solution casting [63].

Surface chemical modification of polysaccharide nanoparticles is another way to decrease their surface energy and disperse them in organic liquids of low polarity. It generally involves reactive hydroxyl groups from the surface. Experimental conditions should avoid media swelling and the peeling effect of surface-grafted chains inducing their dissolution in the reaction medium. The chemical grafting has to be mild in order to preserve the integrity of the nanoparticle. Tunicin microcrystals have been stabilized in tetrahydrofuran (THF) by a partial silylation of their surface [64]. The preparation of bacterial cellulose nanocrystals topochemically trimethylsilylated was also reported [63]. Resulting nanoparticles were dispersed in acetone to process nanocomposites with a cellulose acetatebutyrate matrix. Sterically stabilized aqueous rod-like cellulose microcrystals suspensions were originally prepared by the combination of HCl hydrolysis, oxidative carboxylation and grafting of poly(ethylene glycol) (PEG) having a terminal amino group on one end using water soluble carbodiimide [65]. The PEG-grafted microcrystals displayed drastically enhanced dispersion stability evidenced through resistance to addition of 2 M sodium chloride. They also showed ability to redisperse into either water or chloroform from the freeze-dried state. Alkenyl succinic anhydride (ASA) can be used for acylating the surface of cellulose nanocrystals. ASA is widely used as a sizing agent in papermaking, where ASA is applied to pulp fibers in aqueous systems. Surface chemical modification of crab shell chitin whiskers [66], waxy maize starch nanocrystals [67] and tunicin whiskers [68] with ASA was reported. The acylated whiskers were found to disperse in medium- to low-polarity solvents. It was shown that by controlling the heating time, whiskers with different dispersibility could be obtained. Nogi *et al.* [69] and Ifuku *et al*. [70] were among the first to use acetylated cellulosic nanofibers in the preparation of reinforced clear plastic.

**Figure 2:**
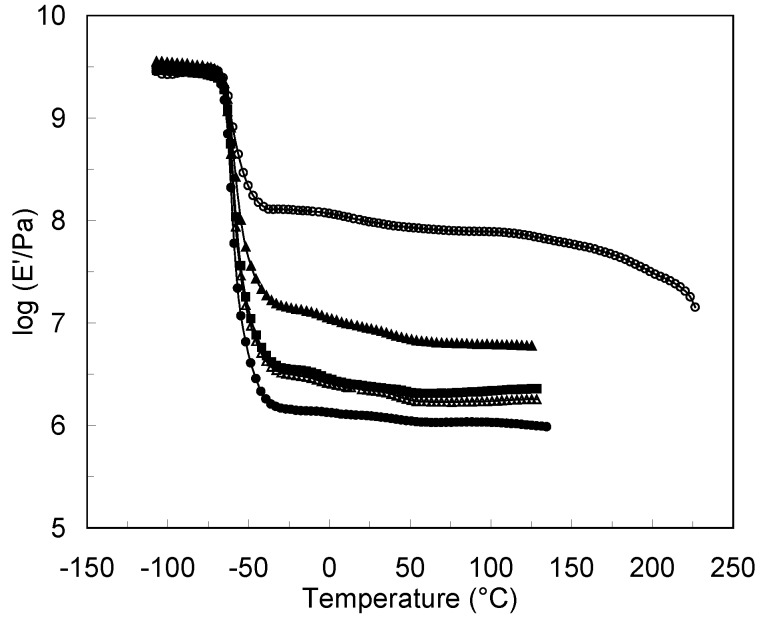
Logarithm of the storage tensile modulus E’ *vs.* temperature at 1 Hz for 10 wt % crab shell chitin whiskers reinforced natural rubber (NR) composites: unfilled NR matrix (●), unmodified whiskers (Ο), whiskers modified with alkenyl succinic anhydride (▲), whiskers modified with phenyl isocyanate (Δ), and whiskers modified with isopropenyl-α-α’-dimethylbenzyl isocyanate (■) (66).

The matrix/filler and filler/filler interactions affect the mechanical behavior of the polysaccharide nanocrystals reinforced nanocomposites. Classical composite science tends to privilege the former as a fundamental condition for optimal performance. In polysaccharide nanocrystals based composite materials the opposite trend is generally observed when the materials are processed via casting/evaporation method [25,66]. The highest the affinity between the polysaccharide filler and the host matrix is the lower the mechanical performances are (Figure 2). This unusual behavior is ascribed to the originality of the reinforcing phenomenon of polysaccharide nanocrystals resulting from the formation of a percolating network thanks to hydrogen bonding forces.

However, the relation between the polymer/nanocrystals affinity and the mechanical performance should be considered with caution. For instance, if we use an apolar matrix like polyethylene (PE) or polypropylene (PP) it is difficult to achieve good dispersion and the agglomeration of nanoparticles takes place leading to poor mechanical performance. The previous sentence is only valid when using a well dispersed nanocrystals liquid suspension for the processing of the composite.

Preparation of stable cellulose whiskers suspensions in dimethylformamide (DMF) [71,72], and dimethyl sulfoxide (DMSO) or *N*-methylpyrrolidine (NMP) [73] without either addition of a surfactant or any chemical modification was also reported. From DMF, tunicin whiskers reinforced POE plasticized with tetraethyleneglycol dimethyl ether (TEGDME) were prepared by casting and evaporation of DMF [45]. Cross-linked nanocomposites were also prepared by dispersing cellulose nanocrystals in a solution of an unsaturated linear polycondensate, addition of a photo-initiator, casting, evaporating the solvent and UV-curing [74].

### 2.4. Long chains grafting

**Figure 3:**
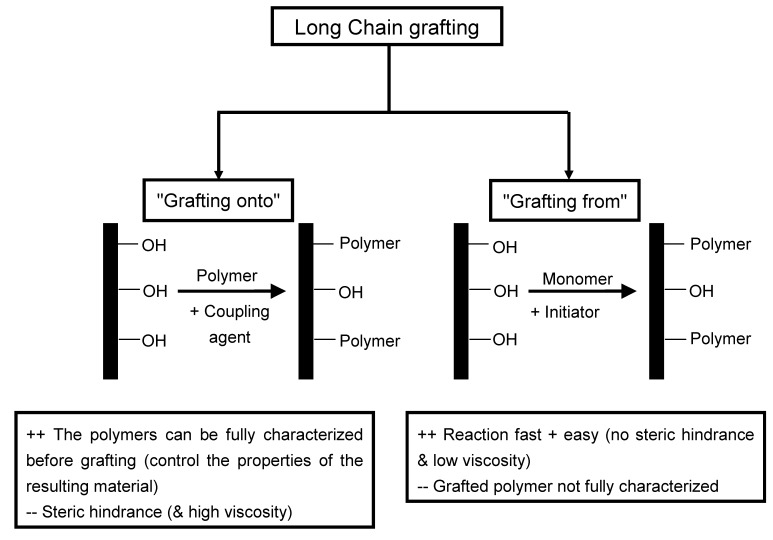
Schematic representation of the "grafting onto" and "grafting from" approaches.

Long chain surface chemical modification of polysaccharide nanoparticles consisting in grafting agents bearing a reactive end group and a long "compatibilizing" tail was also reported in the literature. Two strategies can be envisaged, *viz.* the "grafting onto" and "grafting from" approaches (Figure 3). The general objective is of course to increase the apolar character of the nanoparticle. In addition, it can yield some extraordinary possibilities. The surface modifications can act as binding sites for active agents in drug delivery systems or for toxins in purifying and treatment systems. These surface modifications may also be able to interdiffuse, upon heating, to form the polymer matrix phase. The covalent linkage between reinforcement and matrix will result in near-perfect stress transfer at the interface with exceptional mechanical properties of the composite as a result.

**Figure 4:**
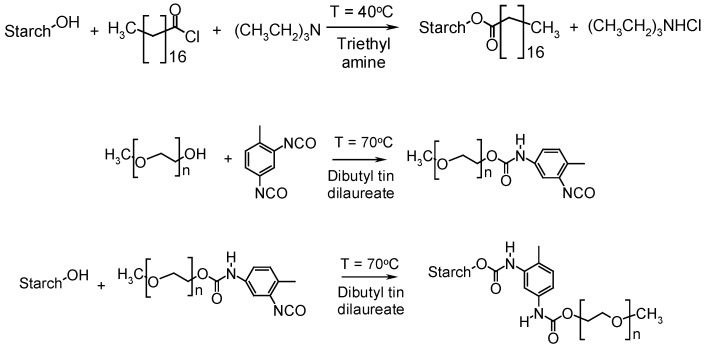
Reaction schemes for the grafting of (a) stearates and (b) PEGME to the starch nanocrystal surface [75].

Thielemans *et al.* [75] reported the chemical modification of starch-derived nanocrystals with poly(ethylene glycol) methyl ether (PEGME) and stearic acid chloride. The former used toluene 2,4-diisocyanate (2,4-TDI) to graft the PEGME to the starch surface. The reaction schemes are shown in Figure 4. Successful surface modification was confirmed using Fourier Transform infrared (FTIR) and X-ray photoelectron (XPS) spectroscopies, and differential scanning calorimetry (DSC) as well as contact angle measurements. An efficient and complete surface coverage was confirmed while the starch crystalline structure was not affected. X-ray diffraction showed extensive crystallization of the stearate moieties grafted at the starch nanoparticles surface, forming a crystalline hydrophobic shell around the hydrophilic starch nanocrystal. Both modifications exhibit a large effect on the individualization of the nanocrystals due to reduced hydrogen bonding and polar interactions between the individual particles. Starch nanoparticles were also successfully grafted with poly(tetrahydrofuran) (PTHF), poly(caprolactone) (PCL), and poly(propylene glycol) monobutyl ether (PPGBE) chains using toluene 2,4-diisocyanate as a linking agent [76]. TEM observations of modified starch nanocrystals showed either the individualization of nanoparticules or the formation of a film, depending on the polymer used. It was shown that grafting efficiency decreased with the length of polymeric chains, as expected.

Nanocomposite materials were processed from PCL-grafted ramie cellulose whiskers and PCL-grafted waxy maize starch nanocrystals using the "grafting onto" [77] approach using isocyanate-mediated reaction. It consists in directly grafting the existing polymer on the surface on the nanoparticles with the use of a coupling agent (Figure 3). The main advantage of this technique is that the polymers can be fully characterized before grafting and then it is possible to control the properties of the resulting material. The main drawback is due to steric hindrance and high viscosity of the medium. The polysaccharide nanocrystals were never dried before grafting but solvent exchanged from water to toluene. For that, aqueous suspension with desired amount of cellulose or starch nanocrystals (1 wt %) was solvent exchanged to acetone and then to dry toluene by several successive centrifugation and redispersion operations. Sonication was performed after each solvent exchange step to avoid aggregation. However, the suspension in toluene was not stable in time. Grafted polymeric chains were found to form a crystalline structure at the surface of the nanoparticles as evidenced from X-ray diffraction and differential scanning calorimetry experiments. Nanocomposite films were processed from both unmodified and PCL-grafted nanoparticles, and PCL as matrix using a casting/evaporation technique. It was shown that mechanical properties of resulting films were notably different. Compared to unmodified nanoparticles, the grafting of PCL chains on the surface resulted in lower modulus values but significantly higher strain at break. This unusual behavior clearly reflects the originality of the reinforcing phenomenon of polysaccharide nanocrystals resulting from the formation of a percolating network thanks to chain entanglements and co-crystallization.

**Figure 5:**
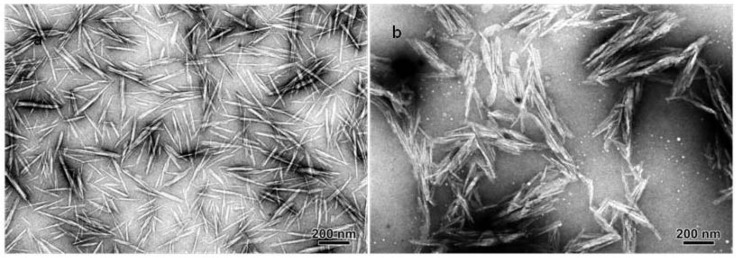
Transmission electron micrograph of ramie cellulose whiskers: (a) ungrafted and (b) recovered after ROP/grafting reactions and Soxhlet extraction [78].

The "grafting from" approach was also used to prepare PCL-grafted cellulose whiskers [78]. The main differences with the "grafting onto" approach are reported in Figure 3. It consists in mixing the nanoparticles with the monomer and an initiator. The polymer is growth directly on the surface of the nanoparticle. The main advantage of this approach is that the reaction is fast and easy because there is no steric hindrance and the viscosity of the reaction medium remains low. The main drawback is that the grafted polymer is not fully characterized. PCL was grafted by Sn(Oct)_2_-catalyzed ring-opening polymerization (ROP). The grafting efficiency was evidenced by the long-term stability of suspension of PCL-grafted cellulose nanocrystals in toluene. After PCL grafting, the structural and morphological integrity of the cellulose nanocrystals didn’t appear to have been affected by Sn(Oct)_2_-catalyzed polymerization and grafting as shown by comparing the TEM observation of ungrafted and grafted nanoparticles (Figure 5). 

The nanocrystals were less individualized than native ones and were believed to aggregate as a result of sulfate groups being removed from their surface. Furthermore, the presence of hydrophobic PCL chains on the nanocrystals likely triggers the particle aggregation upon drying. Nanocomposites with high filler content were prepared from neat and PCL-grafted cellulose nanocrystals and high molecular weight PCL as matrix using a casting/evaporation technique from dichloromethane. A similar approach was used to graft PCL onto MFC via ring-opening polymerization [79]. From thermogravimetric analysis (TGA) measurements, the fraction of grafted PCL was estimated to 16%, 19% and 21% depending on the amount of free initiator to the system. The non-isothermal crystallization shows that a much longer time is required for complete crystallization of PCL grafted to the surface than for free PCL chains. It was ascribed to the lower mobility of the PCL chain when anchored to the MFC surface.

PCL-grafted cellulose nanocrystals prepared using the "grafting from" strategy were thermoformed to produce sheets with good mechanical properties [80]. It was ascribed to the possibility of entanglement of grafted PCL chains during the melting process of thermoforming. Furthermore, the shielding of PCL grafted onto the surface of the nanoparticles contributed to impart high hydrophobicity to the composite. However, the investigation of these materials was limited due to the lack of effective means to determine the number and length of grafted PCL chains accurately, which is inherent to this strategy. Similar results were obtained for injection-molded PCL-grafted crab shell chitin whiskers [81].

PCL was grafted to the surface of starch nanocrystals via micro-wave-assisted ROP [82,83,84]. The ensuing nanoparticles were incorporated into a poly(lactic acid) (PLA) matrix by a casting/evaporation method using dichloromethane [82,83] or waterborne polyurethane matrix [84]. Both the elongation at break and strength of PLA were enhanced when adding 5 wt % nanoparticles. The grafted PCL chains were found to improve the association of starch nanocrystals with the PLA matrix and to form an interfacial layer able to transfer stress. The rubbery PCL phase provided enough flexibility to improve the ductility of the material but decreased the Young’s modulus value. When increasing the filler content, PCL-grafted nanoparticles self-aggregated as bigger agglomerates and inhibited the reinforcing capability of the nanoparticles.

Cellulose whiskers were also surface-grafted with PCL via microwave-assisted ring-opening polymerization yielding filaceous cellulose whisker-graft-PCL nanocrystals which were incorporated into PLA as matrix [83]. Epoxy functionality was introduced onto the surface of cellulosic nanoparticles by oxidation by cerium (IV) followed by grafting of glycidyl methacrylate [85]. The length of the polymeric chain was varied by regulating the amount of glycidyl methacrylate. The surface of cellulose whiskers was also chemically modified by grafting organic acid chlorides presenting different lengths of the aliphatic chain by an esterification reaction [86]. These functionalized nanoparticles were extruded with low density polyethylene (LDPE) to prepare nanocomposite materials. Cellulose whiskers reinforced waterborne polyurethane nanocomposites were synthesized via *in situ* polymerization using casting/evaporation technique [87]. The grafted chains were able to form a crystalline structure on the surface of the nanoparticles and induce the crystallization of the matrix. Cellulose nanoparticles were modified with n-octadecyl isocyanate (C_18_H_37_NCO) using two different methods with one consisting of an *in situ* solvent exchange procedure [88]. Phenol was also enzymatically polymerized in the presence of TEMPO-oxidized cellulosic nanoparticles to prepare nanocomposites under ambient conditions [89].

### 2.5. Extrusion and impregnation

**Figure 6:**
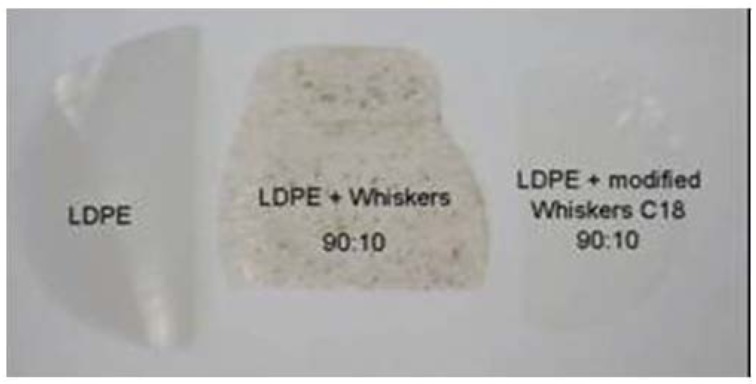
Photographs of the neat LDPE film and extruded nanocomposite films reinforced with 10 wt % of unmodified and C18 acid chloride-grafted cellulose whiskers [86].

Very few studies have been reported concerning the processing of polysaccharide nanocrystal- reinforced nanocomposites by extrusion methods. The hydrophilic nature of polysaccharides causes irreversible agglomeration during drying and aggregation in non-polar matrices because of the formation of additional hydrogen bonds between amorphous parts of the nanoparticles. Therefore, the preparation of cellulose whiskers reinforced PLA nanocomposites by melt extrusion was carried out by pumping the suspension of nanocrystals into the polymer melt during the extrusion process [90]. An attempt to use PVA as a compatibilizer to promote the dispersion of cellulose whiskers within the PLA matrix was reported [91]. Organic acid chlorides-grafted cellulose whiskers were extruded with LDPE [86]. The homogeneity of the ensuing nanocomposite was found to increase with the length of the grafted chains (Figure 6).

An attempt to use a recently patented concept (Dispersed Nano-Objects Protective Encapsulation - DOPE process) intended to disperse carbon nanotubes in polymeric matrices was reported. Physically cross-linked alginate capsules were successfully formed in the presence of either cellulose whiskers or microfibrillated cellulose [92]. The ensuing capsules have been extruded with a thermoplastic material.

Another possible processing technique of nanocomposites using cellulosic nanoparticles in the dry state consists in the filtration of the aqueous suspension to obtain a film or dried mat of particles followed by immersion in a polymer solution. The impregnation of the dried mat is performed under vacuum. Composites were processed by filling the cavities with transparent thermosetting resins such as phenol formaldehyde [93,94,95], epoxy [96], acrylic [94,97,98] and melamine formaldehyde [99]. Nonwoven mats of cellulose microfibrils were also used to prepare polyurethane composite materials using film stacking method [100].

Water-redispersible nanofibrillated cellulose in powder form was recently prepared from refined bleached beech pulp by carboxymethylation and mechanical disintegration [101]. However, the carboxymethylated sample displayed a loss of crystallinity and strong decrease in thermal stability limiting its use for nanocomposite processing.

### 2.6. Electrospinning

Electrostatic fiber spinning or "electrospinning" is a versatile method to prepare fibers with diameters ranging from several microns down to 100 nm through the action of electrostatic forces. It uses an electrical charge to draw a positively charged polymer solution from an orifice to a collector. Electrospinning shares characteristics of both electrospraying and conventional solution dry spinning of fibers. The process is non-invasive and does not require the use of coagulation chemistry or high temperatures to produce solid threads from solution. This makes the process particularly suited to the production of fibres using large and complex molecules.

Bacterial cellulose whiskers were incorporated into POE nanofibers with a diameter of less than 1 µm by the electrospining process to enhance the mechanical properties of the electrospun fibers [102]. The whiskers were found to be globally well embedded and aligned inside the fibers, even though they were partially aggregated. Electrospun polystyrene (PS) [103], PCL [104] and PVA [105] microfibers reinforced with cellulose nanocrystals were obtained by electrospinning. Nonionic surfactant sorbitan monostearate was used to improve the dispersion of the particles in the hydrophobic PS matrix.

### 2.7. Multilayer films

The layer-by-layer assembly (LBL) is a method by which thin films particularly of oppositely charged layers are deposited. Thin film LBL assembly technique can also be utilized for nanoparticles. In general the LBL assembly is described as sequential adsorption of positive or negative charged species by alternatively dipping into the solutions. The excess or remaining solution after each adsorption step is rinsed with solvent and thus a thin layer of charged species on the surface ready for next adsorption step is obtained. There are many advantages of the LBL assembly technique and these include simplicity, universality and thickness control in nanoscale. Further the LBL assembly process does not require highly pure components and sophisticated hardware. For almost all-aqueous dispersion of even high molecular weight species, it is easy to find an LBL pair that can be useful for building thin layer. In each adsorption step, either a monolayer or a sub monolayer of the species is obtained and therefore the number of adsorption steps needed for a particular nanoscale layer can be founded.

The use of the LBL technique is expected to maximize the interaction between cellulose whiskers and a polar polymeric matrix, such as chitosan [106]. It also allows the incorporation of high amounts of cellulose whiskers, presenting a dense and homogeneous distribution in each layer.

Podsiadlo *et al*. [107] reported the preparation of cellulose whiskers multilayer composites with a polycation, poly-(dimethyldiallylammonium chloride) (PDDA), using the LBL technique. The authors concluded that the multilayer films presented high uniformity and dense packing of nanocrystals. Orientated self-assembled films were also prepared using a strong magnetic film [108] or spin coating technique [109]. The preparation of thin films composed of alternating layers of orientated rigid cellulose whiskers and flexible polycation chains was reported [110]. Alignment of the rod-like nanocrystals was achieved using anisotropic suspensions of cellulose whiskers. Green composites based on cellulose nanocrystals/xyloglucan multilayers have been prepared using the nonelectrostatic cellulose-hemicellulose interaction [111]. The thin films were characterized using neutron reflectivity experiments and AFM observations. More recently, biodegradable nanocomposites were obtained from LBL technique using highly deacetylated chitosan and cellulose whiskers [106]. Hydrogen bonds and electrostatic interactions between the negatively charged sulfate groups on the nanoparticles surface and the ammonium groups of chitosan were the driving forces for the growth of the multilayered films. A high density and homogeneous distribution of cellulose nanocrystals adsorbed on each chitosan layer, each bilayer being around 7 nm thick, were reported. Self-organized films were also obtained using only charge-stabilized dispersions of celluloses nanoparticles with opposite charges [112] from the LBL technique.

## 3. Conclusions

Due to their abundance, high strength and stiffness, low weight and biodegradability, nanoscale polysaccharide materials serve as promising candidates for the preparation of bionanocomposites. A broad range of applications of these nanoparticles exists, even if a large number of unknowns remain to date. Tens of scientific publications and experts show its potential even if most of the studies focus on their mechanical properties as reinforcing phase and their liquid crystal self-ordering properties. The homogeneous dispersion of cellulosic nanoparticles in a polymer matrix is challenging. In addition, it is worth noting that there are many safety concerns about nanomaterials, as their size allows them to penetrate into cells and eventually remain in the system. There is no consensus about categorizing nanomaterials as new materials.
